# Lower Back Pain as an Occupational Hazard Among Ugandan Health Workers

**DOI:** 10.3389/fpubh.2021.761765

**Published:** 2021-12-01

**Authors:** Michael Aleku, Kevin Nelson, Anne Abio, Michael Lowery Wilson, Herman Lule

**Affiliations:** ^1^Faculty of Clinical Medicine and Dentistry, Kampala International University, Kampala, Uganda; ^2^Injury Epidemiology and Prevention (IEP) Research Group, Turku Brain Injury Centre, Turku University Hospital and University of Turku, Turku, Finland; ^3^Heidelberg Institute of Global Health, Heidelberg, Germany; ^4^Department of Surgery, Directorate of Research and Innovations, Kampala International University, Kampala, Uganda

**Keywords:** injury, occupational health, sub saharan Africa, pain, backache

## Abstract

**Background:** Lower back pain is a public health concern affecting 70–85% of the world's population. There is paucity of published data on the prevalence, disability and risk factors for lower back pain among health workers in Uganda.

**Objective:** To determine the frequency rate (note that is it implicit that frequency is a rate like incidence so including rate seems redundant here. This is bounded by zero and infinity. In contrast, prevalence is bounded by 0 and 1 and is thus a proportion not a rate) of lower back pain and its associated risks amongst health professionals in the Arua District of Uganda.

**Methods:** Cross-sectional descriptive study of 245 consecutive participants conducted during February-April 2020. We stratified risks as individual or work related and analyzed the data using IBM SPSS version 25. Chi-square was used to measure the significance of association between categorical variables at 95% confidence interval, regarding a *p* ≤ 0.05 as significant.

**Results:** The mean age of participants was 40.87 years ± 8.74 (SD), with female predominance (69.8%). Majority were either general nurses or midwives (64.9%) and more than half had practiced for over 6–10 years. The frequency rate of lower back pain was 39.6% (*n* = 97). Individual factors associated with LBP were; cigarette smoking (*X*^2^ = 33.040; *P* ≤ 0.001), alcohol consumption (*X*^2^ = 13.581; *P* ≤ 0.001), age (*X*^2^ = 14.717; *P* = 0.002), and female gender (*X*^2^ = 4.802; *P* = 0.028). The work related factors significantly associated with lower back pain were: being a nurse/midwife (*X*^2^ = 9.829; *P* = 0.007), working in the outpatient department (*X*^2^ = 49.752; *P* ≤ 0.001), bending (*X*^2^ = 43.912; *P* ≤ 0.001), lifting (*X*^2^ = 33.279; *P* < 0.001), over standing (*X*^2^ = 40.096; *P* ≤ 0.001), being in awkward positions (*X*^2^ = 15.607; P= < 0.001), and pushing patients (*X*^2^ = 21.999; *P* ≤ 0.001).

**Conclusion:** The frequency rate of low back pain was high amongst health workers and its main associated individual and work related factors could have been prevented. Health workers should strike a balance between caring for their personal back-health and meeting clients' needs while manually handling patients. Ergonomic structuring, job organization, back health care courses and use of assistive equipment could reduce such occupational hazards in our low resourced settings.

## Introduction

Lower back pain (LBP) is a global problem of public health importance, affecting 70–85% of the world's population (1). It is a common cause of work-related disability (2). According to Hartvigsen et al. the annual prevalence of lower back pain ranges from 15 to 45%, with a point prevalence averaging 30%. In the United States (US), back pain is the most common cause of activity limitation in people under the age of 45 years and is considered the second most frequent reason for visits to a physician (3). It is also ranked the fifth leading cause of admission to a hospital and the third leading cause of surgical procedures. As such, two percent of the US workforce is compensated for back injuries annually (3). In addition, LBP is reported to be the second leading cause of work absenteeism and results in lost productivity more than any other medical condition (4, 5). According to Hartvigsen et al. (2), the direct and indirect costs attributable to LBP are enormous in terms of loss of quality of life, productivity and employee absenteeism. This condition is thus the single largest contributor to musculoskeletal disability worldwide (6). In the US, it is estimated that over 80 billion US dollars are spent on LBP annually, accounting for over 156 million lost working days and 5.2 million disabilities of which 2.6 million are permanent (2).

In the United Kingdom, it is estimated that 116 million productive days are lost due to LBP and the resulting economic cost is estimated at 12 billion GBP annually (7) whereas in Europe, the direct costs related to LBP are estimated at 7,000 Euros per person per year in Germany (8) and 740 Euros annually in Sweden (9). There is a paucity of retrievable research evidence on economic cost of LBP in Africa. The financial impact of LBP is presumed enormous on the African continent due to its fragile health systems with limited human and infrastructural resource capacity, amidst a dual burden of infectious and non-communicable diseases. The sick leaves related to LBP exert strain on services and staff coverage with absenteeism being identified as an essential indicator of LBP related disability (10). In recent years, medical consultations due to LBP have increased significantly and LBP can be considered a “twentieth-century healthcare disaster” (2).

To date, several studies have revealed a number of risk factors associated with LBP in the general population such as: advanced age, alcohol and drug abuse, family history, gender, level of activity, obesity, poor posture and alignment, smoking; occupational factors such as prolonged standing and sitting, previous back injury plus psychological and social factors (1, 6, 11). Understanding the risk factors for LBP amongst specific population groups is key to guide preventive polices which are tailored to one's occupation. The health sector workforce is one of such special groups that deserves utmost attention, being a core building block for a functional health system. As such, hospital workers have been shown to have higher rates of LBP compared to the general population due to the physical and emotional factors such as stress involved in their occupation (2, 5).

The main occupational risk factors for LBP amongst health workers include: lifting and moving patients, frequent twisting and bending, sustained postures, improper ergonomics of work environment, anxiety, depression, stress, poor job satisfaction, shortage of staff and poor working conditions amongst others (4, 5, 12). However, there is a paucity of published data on the proportion and risks for LBP amongst health workers in low income countries despite resource constraints such as lack of assistive equipment for lifting patients, which requires manual in-patient transfers. Such heavy lifting could lead to physical injuries for instance, involving the vertebral discs, culminating in LBP and restricted movement.

In the end, the limited range of physical movements which results from LBP, can be associated with psychological distress that further intensify the pain, depending on one's coping strategy (13, 14). Indeed, LBP has been linked to psychosocial stress (15), for which cognitive behavioral therapy is being proposed as adjunct in its management (16, 17). According to Bogduk (18), when LBP persists, there is a tendency for the brain activity to switch away from pain circuits to emotional circuits, raising anxiety. Thus, the physical work challenges such as lifting patients manually in low income countries could potentially aggravate the existing psychosocial stress already posed by COVID-19 infections amongst health workers (19), yet mindfulness-based stress reduction (20) is an under-developed field of LBP control and less studied in low income settings compared to higher income (17, 21).

Despite this, few studies have been conducted on LBP amongst health workers in low income countries in the African region which implies an under-estimation of the global burden of LBP. In a systematic review on prevalence of chronic LBP worldwide, only one of the 25 original population-based cross-sectional studies were from Africa (Nigeria), the rest of studies being largely from Europe, Americas, and Asia (22). This indicates how this subject matter is under-researched on the African continent. Inadequate attention on this topic in Africa may be attributed to the outsized impact of infectious diseases which has resulted in the shift of funding priorities within health research to this area (23). According to Morris et al. (23), the mean point prevalence of LBP amongst the adult population in Africa is estimated at 39% whereas chronic LBP ranges from 51 to 63%. In addition, hospital-based statistics show that LBP accounts for 30–40% of visits to rheumatologists in Africa (24) and that much of this burden has been linked to poor back care ergonomics and unavailability of lifting equipment (25). However, the these studies have been disproportionately conducted in South Africa and West Africa (Nigeria) (23) while underrepresented in East Africa.

In Uganda, the point prevalence of LBP amongst health workers was last estimated more than a decade ago at 20% in a hospital-based study at the National Referral Hospital, Mulago (26), partly attributed this to the high levels of perceived stress. Such stress due to LBP is further aggravated by a significant reduction in activities of daily living such as recreation, sleep and sex (27). Ugandan public hospitals have shortages of health workers due to limited health care professionals training capacity and health workforce emigration to the private sector and overseas (28). This in turn, has resulted in increased workloads for staff in public health facilities, thus predisposing them to LBP. Consequently, the impact of absenteeism from duty due to LBP of the already understaffed health workforce in Uganda, underscores the need to better address this problem. This study is therefore aimed at generating current data on the frequency rate and the specific risk factors for LPB among health workers in the Arua District of Uganda. We defined LBP as self-reported pain at the time of the study, that lasts for ≥ 3 months in the area between the twelfth ribs and gluteal folds. The 3-months period case definition was chosen so to minimize recall bias and had been validated in previous studies (23).

## Methods

### Study Design

This was a cross-sectional descriptive study of 245 consecutively recruited health professionals who consented to the study during 1st February 2020–30th April 2020. A multi-center prospective randomized control trial would have provided more generalizable results, giving all endangered health workers equal chance to participate in the study. However, the cost-prohibitive nature this methodology and the ethical implications of consenting participants to exposure variables leading to the outcome of LBP in the present study were key constraints. On the other hand, the low accuracy of paper-based data capture health systems in Ugandan settings would significantly impact data quality for a retrospective cohort (29). Consecutive sampling while sub-optimal, was deemed suitable for our study population that was presumed to be fairly homogeneous with respect to the underrepresented group of health workers in resource limited low-income settings, to offer a narrower but clear generalizability in a context described by Jager et al. (30).

### Study Settings

The study was conducted amongst health workers at Arua Regional Referral Hospital located in the Arua District of Uganda (03′001′10″N; 30′054′45″E). This is a 272 bed capacity, public tertiary and teaching hospital for Arua School of Nursing which serves 8 districts in Northern Uganda and referrals from neighboring parts of southern South Sudan and the Democratic Republic of Congo.

### Eligibility Criteria

Any health worker at the Arua Regional Referral Hospital who was willing to participate in the study. We excluded health workers with a documented history of physical injuries such as those resulting from road traffic crash, falls from height and those with congenital musculoskeletal deformities such as kyphosis and scoliosis that were presumed to influence the dependent variable (LBP) (31, 32).

### Sample Size Determination

To determine the frequency rate of LBP amongst health workers at the Arua Regional Referral Hospital, we used the Kish-Leslie formula (33) to determine the sample size. Based on a prevalence of LBP of 20% reported in a previous study in Uganda (26), a margin error of 5% and a standard normal deviation of (1.96) corresponding to the 95% confidence interval; the sample size that is required for validating the findings was 245 participants. To probe the potential risk factors for LBP, it was deemed unnecessary to calculate a sample size for the purposes of demonstrating a valid association between LBP with each of the individual variables of interest, since this exploratory study intended to report findings that could allow for defining possible associations with the final result (LBP) but not casual inference.

### Study Procedure

Each consenting participant was asked to complete a pre-designed survey questionnaire to collect information on the independent socio-demographic variables, self-reported LBP as the dependent variable and its presumed risk factors as the other independent variables. The independent variables were stratified into two categories: personal or occupational (work-related). Personal variables included age, sex, body mass index, cigarette smoking and alcohol consumption whereas occupational factors included ergonomic structuring, job organization, department, cadre of the health personnel, ergonomics of the work environment, working hours and availability of assistive equipment to lift patients. All these factors had been previously found in the literature to be associated with LBP amongst health workers (4, 5, 12, 25).

### Data Processing and Analysis

We analyzed the data using IBM SPSS 25.0 statistics for windows (Armonk, NY, USA, IBM Corp). The participants' age groups were stratified based on the fact that disability amongst patients with LBP had been highest amongst the 30–50 years age group. Galukande et al. (27). Percentages were computed to determine the frequency rate of LBP. Cross tabulation and Chi-square tests were performed to screen for potential associations with the main outcome variable LBP. Chi-square and correlation tests are known precursor to multivariate analyses in the exploratory research (34) such as the present study. Logistic regression models resulting from forward-selected stepwise procedures were used for variables with *p* < 0.1 at bivariate analysis, to determine which factors were associated with LBP. The level of significance was set at *P* < 0.05 at the 95% confidence interval.

### Quality Control

The questionnaires were pre-tested amongst staff at Kampala International University Teaching Hospital in a similar setting so to ensure clarity. The questions that showed ambiguity during pre-testing were revisited and accordingly modified. A test-retest reliability coefficient of ≥0.9 was observed and considered to be excellent for this measure.

### Ethical Consideration

Ethical approval was obtained from Kampala International University Western Campus, School of Biomedical Sciences, Faculty of Clinical Medicine and Dentistry (Ref: BMS/0100/151). Following approval, an introductory administrative letter was issued to the participating health facility in the Arua District. Written informed consent was obtained from the participants before their enrollment into the study. The study followed the Uganda National Council for Science and Technology (2014) guidelines on conducting research involving participating human subjects.

## Results

A total of 245 participants responded to the survey with a response rate of 100%. The frequency rate of LBP among the sample was 39.6% (*n* = 97). The mean age of participants was 40.87 (8.74 SD) years with a majority (51.4%) in the 40–49 years age group. The participants were predominantly females (69.8%) and the majority of these were either nurses or midwives (64.9%) with the smallest proportion being doctors (3.7%). More than half of the participants had been in practice for 6–10 years (53.5%) prior to the study. The majority were neither smokers (80.0%) nor did they consume alcohol (70.2%) and around half (50.2%, *n* = 123) were either underweight or had normal weight as shown in Table 1.

**Table 1:** Sociodemographic characteristics of respondents.

**Characteristics**	**Frequency**	**Percent**
**Age**		
<30	33	13.5
30–39	54	22
40–49	126	51.4
50+	32	13.1
**Gender**		
Female	171	69.8
Male	74	30.2
**Cadre**		
Nurse/Midwife	159	64.9
Allied health	77	31.4
Doctor	9	3.7
**Cigarette smoking**		
No	196	80
Yes	49	20
**Alcohol consumption**		
No	172	70.2
Yes	73	29.8
**Years of practice**		
<5	85	34.7
6–10	131	53.5
>10	29	11.8
**BMI**		
**Underweight or normal weight**	123	50.2
Overweight	73	29.8
Obese	49	20

### Individual Factors Associated With Lower Back Pain

Individual factors associated with LBP were cigarette smoking (*X*^2^ = 33.040; *P* < 0.001), alcohol consumption (*X*^2^ = 13.581; *P* < 0.001), age (*X*^2^ = 14.717; *P* = 0.002), and gender (*X*^2^ = 4.802; *P* = 0.028) as shown in Table 2.

**Table 2:** Bivariate analysis of individual factors associated with LBP.

**Variables**	**Ever suffered**		**Chi-square**	***P*-value**
	**No**	**Yes**		
**Age**			14.717	0.002
<30	29 (19.6%)	4 (4.1%)		
30–39	34 (23.0%)	20 (20.6%)		
40–49	65 (43.9%)	61 (62.9%)		
50+	20 (13.5%)	12 (12.4%)		
**Gender**			4.802	0.028
Female	111 (75.0%)	60 (61.9%)		
Male	37 (25.0%)	37 (38.1%)		
**Cigarette smoking**			33.04	<0.001
No	136 (91.9%)	60 (61.9%)		
Yes	12 (8.1%)	37 (38.1%)		
**Alcohol consumption**			13.581	<0.001
No	91 (61.5%)	81 (83.5%)		
Yes	57 (38.5%)	16 (16.5%)		
**Years of practice**			3.673	0.162
<5	58 (39.2%)	27 (27.8%)		
6–10	75 (50.7%)	56 (57.7%)		
>10	15 (10.1%)	14 (14.4%)		
**BMI**			0.033	0.855
Underweight/Normal	63 (42.6%)	60 (61.9%)		
Overweight	48 (32.4%)	25 (25.8%)		
Obese	37 (25.0%)	12 (12.4%)		

Work-related factors which were found to be associated with LBP included department (*X*^2^ = 49.752; *P* < 0.001), bending (*X*^2^ = 43.912; *P* < 0.001), lifting (*X*^2^ = 33.279; *P* < 0.001), over standing (*X*^2^ = 40.096; *P* < 0.001), being in awkward positions (*X*^2^ = 15.607; *P* < 0.001), pushing patients (*X*^2^ = 21.999; *P* < 0.001)and professional cadre (*X*^2^ = 9.829; P = 0.007) as shown in Table 3.

**Table 3:** Bivariate analysis of work related factors associated with LBP.

**Characteristics**	**Ever suffered LBP**		**Chi-square**	***P*-value**
	**No**	**Yes**		
			49.752	<0.001
**Ward/department**			
OPD	62 (41.9%)	36 (37.1%)		
Medical ward	12 (8.1%)	13 (13.4%)		
Surgical	12 (8.1%)	12 (12.4%)		
Theatre	3 (2.0%)	21 (21.6%)		
Maternity	46 (31.1%)	3 (3.1%)		
Orthopedic	13 (8.8%)	12 (12.4%)		
**Cadre**			9.829	0.007
Nurse/midwife	97 (65.5%)	62 (63.9%)		
Allied health	50 (33.8%)	27 (27.8%)		
Doctor	8 (8.2%)	1 (0.7%)		
**Working hours**			0.033	0.855
<8 h	75 (50.7%)	48 (49.5%)		
>8 h	73 (49.3%)	49 (50.5%)		
**Bending/twisting**			43.912	<0.001
Yes	13 (8.8%)	44 (45.4%)		
No	135 (91.2%)	53 (54.6%)		
**Lifting**			33.279	<0.001
Yes	37 (25.0%)	60 (61.9%)		
No	111 (75.0%)	37 (38.1%)		
**Standing for a long time**			40.096	<0.001
Yes	13 (8.8%)	42 (43.3%)		
No	135 (91.2%)	55 (56.7%)		
**Transferring patients**			6.812	0.009
Yes	98 (66.2%)	48 (49.5%)		
No	50 (33.8%)	49 (50.5%)		
**Awkward positions**			15.607	<0.001
Yes	87 (58.8%)	32 (33.0%)		
No	61 (41.2%)	65 (67.0%)		
**Pushing**			21.999	<0.001
Yes	88 (59.5%)	28 (28.9%)		
No	60 (40.5%)	69 (71.1%)		

Using the data reduction features of SPSS, we identified the variables which were highly correlated with each other such as alcohol and cigarette smoking and re-ran the logistic output using the forward selection feature of SPSS. When the Cox-Snell R square (0.34) and Nagelkerke R square (0.46) were below the minimum threshold value of 2.5 for flagging multi-collinearity [We should probably note that the formula that relates variance inflation to r squared are generally intended to be applicable in a multiple regression rather than logistic models which do not generate r squares but these pseudo r square tests do help support our overall argument since step-wise procedures also throw out some variables that are highly correlated with others], the individual factors (age, gender and cigarette smoking) and work related factors (ward/department, bending or twisting, lifting, and pulling or pushing) remained strongly associated with the presence/absence of LBP with *P* < 0.01 (Table 4).

**Table 4:** Forward stepwise regression analysis of factors associated with LBP.

**Selection**	**Variable**	**Model LL**	**Change in -2 LL**	**Df**	**Sig. of change**
**Individual factors**					
Step 1	Cigarette smoking	–164.474	32.938	1	<0.001
Step 2	Gender	–148.005	19.846	1	<0.001
	Cigarette smoking	–162.099	48.033	1	<0.001
Step 3	Age	–138.082	5.792	1	0.016
	Gender	–146.331	22.29	1	<0.001
	Cigarette smoking	–159.667	48.962	1	<0.001
**Work factors**					
Step 1	Bending or twisting	–164.474	44.11	1	<0.001
Step 2	Bending or twisting	–147.708	38.943	1	<0.001
	Lifting	–142.419	28.365	1	<0.001
Step 3	Bending or twisting	–132.471	24.864	1	<0.001
	Lifting	–137.426	34.774	1	<0.001
	Pulling or pushing	–128.236	16.395	1	<0.001
Step 4	Ward/department	–120.039	13.938	1	<0.001
	Bending or twisting	–123.87	21.6	1	<0.001
	Lifting	–133.638	41.135	1	<0.001
	Pulling or pushing	–126.592	27.043	1	<0.001

*LL, Log Likelihood*.

## Discussion

This study established the frequency rate of LBP to be 39.6%. The individual factors (cigarette smoking, alcohol consumption, age, and female gender) and work-related factors (being a nurse/midwife, working in the outpatient department, bending, lifting, over standing, being in awkward positions, and pushing patients) were significantly associated with LBP.

The frequency rate of lower back pain in this study was 39.6% which is higher than 20% previously reported by a study in Mulago Hospital, Uganda (26). Although close to the African average of 41.9% (35), the higher frequency rate of LBP in the present study might depict a rapid shift in the population aging (1), without a concurrent rise in dedicated resources to address this burden. A cross-sectional survey which analyzed work-related musculoskeletal disorders among nurses in Ibadan in South-West Nigeria found a comparable prevalence of lower back pain at 44.1% (25). However, the frequency of LBP in the present study is lower than 56.2% found in a study conducted among healthcare workers in tertiary health institutions in Sokoto, Nigeria (24). The difference in occurrence rates could be due to the fact that the researchers in Nigeria included the entire state (Sokoto) with a relatively larger sample size. High prevalence of lower back pain are reported in other studies across the globe (2, 5). This high occurrence of lower back pain reduces the efficiency of health workers.

With respect to the individual factors, this study found that age was significantly associated with lower back pain. The highest frequency rate (62.9%) was found among the respondents aged 40–49 years contrary to the study by Mitchell et al. (39) that found no association between age and LBP. However our findings concur with a study amongst health workers in Tunisia which found that occurrence of LBP increased with age, with a peak toward 36–46 years (36). The increased burden of LBP in advanced age is presumed to be related to low bone density and comorbidities in this age group, however in this particular study, this might be rather attributable to prolonged duration of exposure to the manual lifting of patients commensurate to one's working experience. This is however controversial as some studies have found higher prevalence of LBP amongst adolescents, for instance in a systematic review by Steffens et al. (11), although authors warn that such pain could persist and become chronic in old age.

On the other hand, LBP could depend on ones' level of physical activity and seating postures which vary with age, with youth spending more sedentary time on electronic devices such as computers and cell phones. In addition to aging, bone density is also significantly correlated with racial background, with persons of African descent having significantly more dense lumbar bones and thus lower rates of bone mass attrition due to aging (37). Despite this however, the occurrence of LBP in African settings is like to be comparable to well-resourced countries given under reporting (38) due to concerns of higher malnutrition and infectious diseases of the spine such as tuberculosis.

In this study, 61.9% of the respondents with LBP were females and this was significant (is there an associated *p*-value with this?). This is similar to findings of other studies (1, 3, 11). In addition, these findings are comparable to the results found in the Ugandan study of health workers at Mulago hospital that reported 68% occurrence rates for female and 32% for males, respectively. Galukande et al. (26). The high frequency rates in females could be attributed to females predominantly taking on the nursing job roles, inclusive of lifting and transfer of patients in addition to extra occupational workload of women in our cultural settings such as household chores and caring for children. Furthermore, an Australian study suggests that the difference in LBP occurrence may be related to the anatomical, physiological and structural differences between males and females (39). The female hormones such pregnancy induced relaxin and low estrogen levels that are associated with the aging process could aggravate the strain on the bony spine (40).

A higher percentage of nurses in the present study were significantly experiencing LBP as compared to other professionals (63.9 vs.36.1%; *P* = 0.007). A Nigerian study by Kehinde et al. (41) and a systematic review and meta-analysis of risk factors of musculoskeletal disorders in hospital workers reported similar results (5, 24). The higher frequency rate of LBP among nurses in this study could be attributed to the fact that nurses are more involved in the manual handling of patients while carrying out nursing roles. The nurses' job description in Uganda involves direct patient care for instance moving and transferring patients such as from operating theaters to the wards and transporting material and medical devices (26). However, in other African settings, this might be an issue of workload with a tendency to task shifting where nurses take on both doctors' and nursing roles in the event physicians are under staffed (42).

Unlike results of a study by Oliveira et al. (43), which found LBP higher in smokers, this study found otherwise. Majority of the respondents with LBP (61.2%) had no history of smoking. Smoking is presumed to cause LBP through the effect of nicotine on nerves and mineral density but how it exactly exerts these effect is unclear (44). In a systematic review by Furlan et al. (45), it was concluded that heavy lifting and long standing were more reliable predictors of LBP than smoking. Despite conflicting results on the influence of smoking on LBP, it is generally agreed that smoking is harmful to one's health and could either way predispose to LBP (46).

Ward and department, bending and twisting, lifting patients, standing for a long time and pushing patients during transfer were all work related factors found to be significantly associated to LBP amongst health workers. In their study amongst Nigerian health workers, Sikiru (47) found that manual handling during transfer of patients was a major predictor of LBP. In our settings, most patient handling activities are performed in less than ideal space and in sub-optimal time frames. Besides, it's not uncommon to use faulty trolleys such as one with non-functional tires to transfer patient. According to Tinubu et al. (25), repeated biomechanical strain on the musculoskeletal system may eventually lead to the development of LBP. Thus, the increased proportion of participants with LBP in this study could be the result of poor working posture, the incorrect use of lifting techniques and unavailability of manual handling equipment in the health facilities such as job aids. Heavy physical work, sustained position and lifting have been earlier linked to LBP in a Nigerian study (23). In addition, according to Vermani et al. (48) the risk of developing LBP amongst Japanese nurses involved in manual handling of patients was high compared with nurses who were not involved.

This study found that the majority of respondents who had suffered from LBP were working in the outpatients department and the theater. This is in agreement with Manchikanti et al. (49) in USA who found high frequency rate of LBP among health workers in the outpatient department. This may be partly due to the fact that the outpatients department receives all volumes of patients entering the hospitals whereas theater staff transfer critically ill pre-operative and post-operative patients who need lifting or pushing on the trolleys. Poor back care ergonomics and unavailability of lifting equipment have been previously cited as major predisposing factors to LBP among health workers in Africa (25), but minimal has been done to address this issue. In Ugandan public health facilities, inadequate human resource and infrastructural capacity puts extra strain on the fewer health care providers who manually lift these patients (28), but inclusion of manual patient lifting on the nurses' job description during employment further complicates this issue. It is no doubt that nursing staff and midwives who report lifting, and bending while pushing patients such as those from theater report LBP. Other than the physical impact on their health, LPB could be a strong demotivator that can deter individuals from joining the nursing profession in the future, further limiting human resource capacity in the region.

### Study Strengths

The data collection tools were pre-tested for validity. This being a cross-sectional study, the completeness of the questionnaire and overall quality of the data could be easily be controlled.

### Study Limitations

The present study was not without limitations. Firstly,the study established the occurrence of LBP amongst health workers without much emphasis on its severity. Secondly, the self-reported nature of the data collection approach could have been affected by social desirability bias hence distorting the results. As such, participants could have under-reported LBP and certain aspects of their working circumstances for job security concerns such as early retirement on medical grounds or rather over report LBP in such a way as to obtain less strenuous departmental tasks. In addition cross-sectional studies like one described do not give a casual inference. Finally, the psycho-social factors that could contribute to LBP such anxiety, stress, depression, job satisfaction were beyond the scope of this study.

### Conclusion

The frequency rate of LBP amongst health workers was high at 39.6%, mainly affecting those aged 40–49 years, females, and nurses/midwives. In addition, working in the outpatient department, bending/twisting, lifting, and standing for long hours and pushing were associated with a higher risk of LBP. There is urgent need for appropriate assistive devices for manual handling of patients in similar resource constrained settings. Induction courses on lower back care, correct lifting techniques, individual physical exercises and building health public policies for new health worker recruits could mitigate this public health threat. Future research should be multi-center prospective randomized cohort studies to determine the cumulative impact of manual patients handling activities on LBP and the resulting economic implications.

### Data Availability Statement

The original contributions presented in the study are included in the article/Supplementary Material, further inquiries can be directed to the corresponding author.

### Ethics Statement

The studies involving human participants were reviewed and approved by Kampala International University Western Campus, School of Biomedical Sciences, Faculty of Clinical Medicine and Dentistry (Ref: BMS/0100/151). The patients/participants provided their written informed consent to participate in this study.

### Author Contributions

MA, AA, and ML conceived and designed the study, performed literature review, analyzed the data, prepared tables, authored and reviewed drafts of the manuscript, and approved the final manuscript. KN conceived and designed the study, analyzed the data, prepared tables, authored and reviewed drafts of the manuscript, and approved the final manuscript. HL conceived and designed the study, performed literature review, analyzed the data, prepared tables, authored and reviewed drafts of the manuscript, supervised, and approved the final manuscript. All authors contributed to the article and approved the submitted version.

## Funding

This work was funded by the authors and open access publication was supported in part by Frontiers Fee Support Office (DSC-08031593527PRD).

## Conflict of Interest

The authors declare that the research was conducted in the absence of any commercial or financial relationships that could be construed as a potential conflict of interest.

## Publisher's Note

All claims expressed in this article are solely those of the authors and do not necessarily represent those of their affiliated organizations, or those of the publisher, the editors and the reviewers. Any product that may be evaluated in this article, or claim that may be made by its manufacturer, is not guaranteed or endorsed by the publisher.
