# Supplementary material for: Lower Back Pain as an Occupational Hazard Among Ugandan Health Workers
Source: Front Public Health. 2021 Dec 1;9:761765. doi: 10.3389/fpubh.2021.761765 (PMC8671744; doi:10.3389/fpubh.2021.761765)
Supplement: Supplementary file 1 [file Data_Sheet_1.PDF]

CONSENT FORM KEY FACT DOCUMENT

Dear Participant, Lower back pain is a growing concern contributing to time lost while staff are away from duty. This research evaluates the potential of Low Back Pain as an occupational hazard amongst health workers in Arua district of Northern Uganda. The findings will provide current information regarding this burden amongst Ugandan health workers. If you are a health professional in Arua district, you are eligible to participate. You will only be required to provide information require on the questionnaire and have your weight/height taken by investigators. Your participation is entirely voluntary and you can cancel your participation any time you wish doing so. There are no monetary benefits from the study however you will learn current updates on risks of low back pain during the course of the study. Should the investigators notice you have back health problems, referral and linkage to a physiotherapist and spine surgeon will be arranged for you. Apart from you disclosing your health information to the investigators, there are no other major risks of participating in the study. The information you provide will be kept anonymous all the time, including archiving and confidential. Should you have any questions, please contact Kampala International University through Dr. Herman Lule (lule.herman@gmail.com) or the Research and Ethics Committee through Prof. Ssebuufu Robinson (rssebuufu@gmail.com).

CONSENT

I ……………………………………………………………………………………(Initials)

Hereby give consent to the researcher to collect any information from me concerning **prevalence and associated factors of low back pain among health workers in Arua district in Uganda**. I agree also to fill the questionnaire presented to me. I hereby declare that the basis of my consent is the comprehensive explanation of the researcher. I understand the information is purely for research purposes only and it will be kept confidential.

By signing this consent form, I authorize my participation in the study.

Signed …………………………Date ……………………………………………………….

In presence of investigator (Sign)………………………………Date……………………………

In presence of Legal representative (Sig)……………………….Date……………………………
