# Supplementary material for: Lower Back Pain as an Occupational Hazard Among Ugandan Health Workers
Source: Front Public Health. 2021 Dec 1;9:761765. doi: 10.3389/fpubh.2021.761765 (PMC8671744; doi:10.3389/fpubh.2021.761765)
Supplement: Supplementary file 2 [file Data_Sheet_2.PDF]

LOW BACK PAIN RESEARCH QUESTIONNAIRE

This research will assess low back pain as a potential occupational hazard at your workplace. Please answer the following questions. Where there is a choice of responses, please circle the correct response or tick

Section 1: Individual Factors

1) Cadre of the health worker: ………………………
2) What is your age in years?..
3) What is your sex assigned at birth? MALE / FEMALE/OTHER
4) **History of Smoking and Alcohol Consumption**

1. Do you currently smoke cigarettes?	YES	NO
2. Have you ever smoked before?	YES	NO
3. Do you drink alcohol?	YES	NO

5) **Shift Work Details.**

1. How many years have you been practicing?	
2. Which ward are you currently working in?	
3. How long have you worked in this ward?	
4. What other wards have you worked in the last years?	

6) **Employment History**

1. Are you currently on day duty?	YES	NO
2.Are you currently on night duty	YES	NO
3. Are you permanently on day/night duty?	DAY	NIGHT
4. If rotating, how often are you rotated to other wards?	Every 3 months<br><br>Every 6 months<br><br>Yearly<br><br>Specify	

Section 2: Low Back Pain (LBP) History

DEFINITION: Low Back Pain is pain lasting for 3 months or longer in an area between the twelfth ribs and the gluteal folds

7)

1. Have you suffered from LBP in the past 3 months?	YES	NO
4. Did you ever suffer from LBP before working as a health worker?	YES	NO
5. How many days have you been absent from work in the past year due to low back pain?		

8) **What work activities cause your Low back symptoms to recur? Please Tick all that apply.**

1. Bending or Twisting	
2. Lifting	
3. Maintaining a position for long periods of time e.g. standing, sitting, kneeling	
4. Performing manual therapy techniques e.g. massage, mobilization	
5. Performing repetitive tasks	
6. Reaching or working away from the body	
7. Transferring patients	
8. Working in cramped/awkward positions	
9. Pushing or pulling	
10. Other. Please specify:	

9) **What type of low back injury did you incur? What was the current diagnosis?**

1. N/A (No diagnosis)	
2. Degeneration	
3. Ligament Sprain	
4. Muscle Strain	
5. Neuropathy	
6. Vertebral disc involvement	
7. Other: Please specify:	

Section 3: Occupational Factors

10) How long do you spend doing the following activities? Please mark all that apply

ACTIVITIES	HOURS
1.Lifting	
2.Transfers	
3.Bending	
4.Sitting	
5.Standing	

11) What work activity were you doing when you injured your back?

1.Giving Medication	
2. Bending or Twisting	
3. Instructing a patient	
4. Lifting	
5. Mantaining a position for a prolonged period of time. Please specify the posture. E.g. standing, sitting, kneeling or bent over	
6. Performing repetitive tasks	
7. Responding to an unanticipated or sudden movement by a patient	
8. Transferring a patient	
9. Working in an awkward or cramped position	
10. Working when physically fatigued	
11. Pushing or pulling	
12. Other. Please specify:	

12) In the following table are 18 potential job risk factors. On a scale of 0-5; 0 being no problem and 5 being a major problem, please indicate to what extent each risk factor may be implicated in the development of your current low back pain.

JOB RISK FACTOR	0	1	2	3	4	5
1. Performing the same task over and over						
2. Working in the same position for long periods of time						
3. Working a shift with few staff on duty						
4. Bending or twisting your back in an awkward way						
5. Lifting or transferring dependent patients						
6. Continuing to work when injured or hurt						
7. Reaching or working away from your body						
8. Working in awkward or cramped positions						
9. Working near to or at your physical limits.						
10. Not enough rest breaks during the day						
11. Unanticipated sudden movement or fall by a patient						
12. Assisting patient during gait activities						
13. Carrying/lifting or moving heavy materials and equipment						
14. Working with confused or agitated patients						
15. Work schedule (e.g. overtime, on-call, irregular shifts)						
16. Inadequate training in injury prevention						
17. Other. Please specify:						

21) Anthropometry and BMI (The investigator will take your weight and height)

Participant weight in Kg?	
What is your height in meters?	

Thanks for your valuable time and participating in this study
